# Self-Driven Flow Characteristic of Magnetic Nanofluids Under the Magnetic Field

**DOI:** 10.3390/ma19050832

**Published:** 2026-02-24

**Authors:** Jiale Mi, Qiang Yang, Yijun Fu, Binfei Zhan, Zhichao Wang, Meibo Xing

**Affiliations:** 1 School of Environment and Energy Engineering, Beijing University of Civil Engineering and Architecture, Beijing 100044, Chinaxingmeibo@bucea.edu.cn (M.X.); 2 State Key Laboratory of Building Safety and Built Environment, Beijing 100013, Chinawangzh@cnas.org.cn (Z.W.); 3 China Academy of Building Research, Beijing 100013, China

**Keywords:** self-circulation, magnetic nanofluid, magnetic fields, thermomagnetic effect

## Abstract

Against the backdrop of the ever-expanding practical applications of magnetic nanofluids, the self-driven flow and heat transfer characteristics of water-based Fe_3_O_4_ magnetic nanofluids were experimentally investigated under a uniform magnetic field in the closed-loop pipeline system in this work. Specifically, Fe_3_O_4_ nanoparticles were synthesized using the co-precipitation method, and stable magnetic nanofluids with concentrations ranging from 0.025 wt% to 0.150 wt% were prepared using sodium citrate as a dispersant. In the presence of a magnetic field, a closed-loop system that integrates heating and cooling branches was established. Furthermore, the effects of magnetic field strength, temperature difference between the heating and cooling sections, magnetic nanofluid concentration, and pipeline length on the self-circulation flow velocity were discussed, leading to insights into the heat transfer characteristics of the magnetic nanofluid. The results showed that the circulation flow velocity increases with the increase in magnetic field strength, magnetic nanofluid concentration, and temperature difference, while it decreases with the increase in pipeline length. Correspondingly, the heat transfer coefficient between the pipeline wall and the fluid increased significantly with the increase in circulation flow velocity. The priority of factors on the thermomagnetic effect is ranked as magnetic field strength > pipeline length > temperature difference > magnetic nanofluid concentration.

## 1. Introduction

With the increasing demands for energy efficiency and precise temperature control, optimizing heat transfer performance has emerged as a core challenge in areas such as electronics, aviation, and mechanical processing. However, conventional heat transfer fluids, such as water, ethylene glycol, and thermal oils, are limited by their low thermal conductivity and restricted specific heat capacity. This makes it challenging to meet the cooling demands of high-power equipment and extreme operating conditions.

Nanofluids are a class of colloidal suspensions in which nanoscale particles—ranging from metallic constituents to metal oxides and carbon-based compounds—are uniformly dispersed within a base liquid [1]. The high thermal conductivity of nanofluids has attracted considerable interest for enhancing heat transfer performance [2,3,4]. Chandel et al. [5] studied the non-Newtonian pseudoplastic stagnant flow and heat transfer characteristics of Al_2_O_3_ + ZnO + MWCNTs/ethylene glycol ternary nanofluids on an exponentially stretching sheet. Their analysis indicated that key parameters, such as inclined magnetic field, thermal radiation, viscous dissipation, velocity slip, and thermal jump boundary conditions, could significantly affect the performance of nanofluid, suggesting potential applications in aerospace and electronics. Zhou et al. [6] conducted experiments on nanofluids of TiO_2_, SiO_2_, and ZnO, respectively, and studied aspects such as their type, concentration, and particle size. They found that adding nanoparticles would enhance thermal conductivity by 7.19%, 12.94%, and 20.03%, respectively, compared to the pure base fluid. Al-Fatlawi et al. [7] conducted comparative studies on single and mixed nanofluids containing Al_2_O_3_, TiO_2_, and Al_2_O_3_-TiO_2_ mixtures using water and ethylene glycol as base fluids. Their results demonstrated that the heat transfer performance of Al_2_O_3_, TiO_2_, and Al_2_O_3_-TiO_2_ nanofluids improved by 10.04%, 12%, and 18.6%, respectively. Mishra et al. [8] examined the impact of coolants by comparing the performance of carbon nanotube–Al_2_O_3_ (0.1% *w*/*w*) hybrid water-based nanofluid with pure water. The results indicated a maximum temperature decrease of 2.459 K.

Maintaining stable nanofluids with well-dispersed nanoparticles is essential for preserving the suspension’s optimal viscosity. Aggregation may arise from attractive van der Waals interactions or Brownian motion, causing viscosity to rise and reducing fluidity, which in turn degrades heat transfer performance. Particle clustering hinders movement, increases flow resistance, and can lower the effective thermal conductivity. When aggregates surpass a certain size, sedimentation occurs, triggering instability and performance loss associated with viscosity escalation [9]. Surface modification strategies, including the introduction of surfactants or pH tuning, enhance stability by suppressing particle agglomeration and maintaining a favorable viscosity. Attaining an optimal trade-off between thermal conductivity and viscosity thus hinges on enhanced stability, as demonstrated by surfactant-treated nanofluids featuring tailored nanoparticle dispersion [9]. In fact, obtaining stable nanofluids is the key prerequisite for improving their thermal performance [10]. Notably, the stability of nanofluids modified with sodium dodecyl benzene sulfonate (SDBS) and cetyltrimethylammonium bromide (CTAB) is typically achieved at pH values below 6 [11]. Two primary techniques, namely the one-step method [12] and the two-step method [13], are employed for nanofluid preparation based on nanoparticle dispersion mechanisms. Together, these approaches ensure uniform nanoparticle dispersion and stable suspension within the carrier fluid while optimizing thermal and rheological properties to enhance heat transfer performance. To advance nanofluid stability further, physical and chemical strategies are employed. Among physical techniques, ultrasonic treatment and mechanical agitation are the primary methods used to reduce particle size and break up agglomerates [14]. By contrast, chemical approaches involve adjusting the suspension’s pH and introducing various surfactants to tailor the surface properties of nanoparticles [15]. Overall, the described approaches modify the surface properties of nanoparticles and reduce the interparticle forces that facilitate aggregation.

Magnetic nanofluids are magnetic particles suspended in a base fluid. At low concentrations, their flow behavior aligns with Newtonian liquids, and they also exhibit magnetic properties comparable to bulk magnetic materials. The magnetization strength decreases with increasing temperature when magnetic fluids are subjected to an external magnetic field [16]. Under the thermomagnetic effect, in systems exhibiting a temperature gradient, the nanofluid shows higher magnetization at the cooler region than at the hotter region. This difference generates a net magnetic driving force that propels the magnetic nanofluid from the weaker thermal end to the stronger cold end, facilitating self-driven circulation without additional energy consumption [17,18]. Pal [19] designed and fabricated a thermomagnetic pump that drives the flow of ferromagnetic nanofluid solely through temperature and magnetic field gradients. Fumoto et al. [20] conducted research on a miniature thermal transfer device that employed temperature-sensitive magnetic nanofluid. The findings show that by varying the magnetic field strength, the magnet’s position, and the fluid temperature, the flow velocity of the magnetic nanofluid can be regulated, achieving a peak velocity of 20 mm/s in the absence of a pump. Philip et al. [21] developed a multifunctional device equipped with temperature-sensitive magnetic nanofluid, which proved suitable for flow control in micro-particle applications. Lian et al. [22] developed an integrated framework describing fluid flow and heat transfer in temperature-responsive magnetic nanofluids and designed an automatic energy transfer device based on the thermomagnetic effect. This device achieved a stable circulation flow of 0.511 mm/s in a circular channel under a thermal flux density of 5000 W/m^2^, with a magnetic field provided by permanent magnets.

Mei et al. [23] sought to elucidate how several key parameters—such as magnetic induction intensity, nanoparticle mass fraction, electromagnet configuration, tube geometry, and Reynolds number—govern the flow behavior and heat transfer performance of magnetic nanofluids. The results indicate that higher nanoparticle loading, stronger magnetic field intensity, a double-sided staggered electromagnet configuration, and corrugated tube geometries yield more pronounced improvements in heat transfer. Boonkumkrong et al. [24] examined the flow dynamics of Fe_3_O_4_-based nanofluids subjected to alternating magnetic fields. The alternating field enhanced the heat transfer coefficient relative to cases without electromagnetic exposure. Regardless of the presence of a magnetic field, increasing the nanoparticle volume fraction improved heat transfer performance. Dalvi et al. [25] demonstrated sustained flow and self-powered heat transfer of a nanomagnetic nanofluid in a ring-shaped annular cavity, driven exclusively by magnetic forces. They observed that the flow velocity increases when the magnetic field strength grows. Yamaguchi et al. [26] evaluated the energy transfer performance of a magnetically driven cooling device employing binary TSMF under the described configuration. Their experiments confirmed effective long-range heat transport over 5 m, delivering 35.8 W of thermal energy, even when the circulation loop was placed horizontally. Zhu et al. [27] conducted a comprehensive numerical study of the flow, thermal transport, and magnetic force characteristics across varying heat fluxes and remanent magnetizations. When heat flux and remanent magnetization increase simultaneously, convective heat transfer intensifies progressively, with the convective coefficient h spanning approximately 4000 to 9000 W/(m^2^·K). Elevating the heat flux boosts the temperature-gradient-induced force, increasing the imbalance of the magnetic field gradient force and leading to higher flow velocity. Furthermore, at a fixed heat flux, higher remanence enhances both the temperature-gradient-driven force and, to a greater extent, the magnetic field gradient force. Nevertheless, a decrease in circuit temperature elevates viscous losses, resulting in a gradual rise in the circuit’s mean velocity. To improve the thermal performance and enhance the flow-driving force within a circular tube, Zhongwu et al. [28] introduced non-magnetic porous media into the tube. The heat transfer performance was assessed systematically by tracking the fluid velocity and thermal resistance for porous elements with varying lengths and porosities. The results indicate that the maximum flow velocity occurs at a porosity of 80% and a length of 10 mm. B.V.V et al. [29] utilized the thermomagnetic effect to achieve the self-circulation of magnetic nanofluids in an annular device, which provided the device with a maximum temperature drop of 197 °C and the most efficient normalized cooling rate of 29 °C·kW^−1^·m^2^. Yuhiro et al. [30] miniaturized a magnetically driven heat transport device with an inner diameter of 1.54 mm and a flow path length of 1500 to 6000 mm. They experimentally and theoretically demonstrated the promoting effect of flow channel length on the magnetic force-induced flow velocity.

Lian et al. [17] proposed a dual magnetic pump loop that integrates permanent magnets, heating elements, heat exchangers, and a temperature-responsive magnetic nanofluid. Numerical simulations showed that adjusting the external magnetic field and the nanofluid temperature can effectively govern the energy transfer process. The flow velocity of the magnetic nanofluid in the dual-pump configuration exceeds that in the single-pump setup by more than twofold. Mehdi et al. [31] employed a two-phase mixture model to examine the thermohydraulic behavior of a temperature-responsive magnetic nanofluid circulating in a circular loop subjected to a magnetic field. Numerical results revealed that increasing the remanent flux density of the permanent magnet and lowering the heat sink temperature enhances the Nusselt number at the heat source cross-section. Xuan et al. [32] applied thermomagnetic convection for cooling hot chips. Their results demonstrated that as the thermal load increased, enhanced thermomagnetic convection facilitated a higher heat discharge rate, ultimately achieving a cooling capacity of 5 W and a significant reduction in the chip’s surface temperature. Ghadiri M. et al. [33] enhanced the overall performance by introducing a 3 wt% magnetic nanofluid into the collector of a photovoltaic–thermal (PVT) system and by applying an alternating magnetic field. Moreover, the distinctive behavior of magnetic nanofluids leads to changes in their rheological and thermophysical properties when exposed to an external magnetic field. Numerical studies [34,35] suggest that the thermomagnetic effect is more pronounced at smaller scales.

Current research indicates that the factors affecting magnetic nanofluids in the thermomagnetic effect primarily focus on qualitative studies of heating power and non-uniform magnetic fields. Other influencing factors require further investigation. This article primarily examines the experimental effects of magnetic field strength, heating temperature, and the characteristic parameters of magnetic nanofluids on the thermomagnetic convection of magnetic nanofluids. Specifically, magnetic nanofluids were prepared using a chemical co-precipitation method, and a closed-loop circulation system was designed to achieve magnetic-controlled self-circulation of these fluids within the pipeline, based on the principles of thermomagnetic effects. The effects of magnetic field strength, magnetic nanofluid concentration, temperature difference between the hot and cold ends, and pipeline length on the self-circulation flow characteristics and heat transfer of magnetic nanofluids were investigated under the influence of an external magnetic field.

## 2. Materials and Methods

### 2.1. Preparation of Magnetic Nanofluid

**Figure 1:**
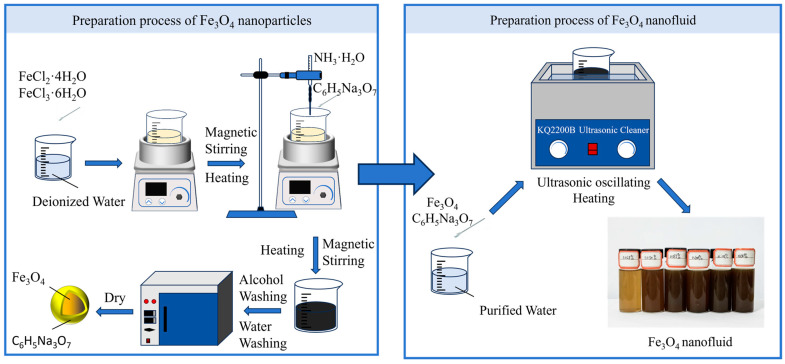
Preparation process of Fe_3_O_4_ magnetic nanofluid.

Fe_3_O_4_ nanoparticles were synthesized using the chemical co-precipitation method, as illustrated in Figure 1. First, 1.98 g of FeCl_2_·4H_2_O and 1.35 g of FeCl_3_·6H_2_O were mixed in 150 mL of deionized water. The solution was heated to 50 °C using a magnetic stirrer set to a stirring speed of 800 r/min. When the temperature reached 50 °C, 15 mL of 25% aqueous ammonia was added to adjust the solution pH to 10. Thereafter, 0.5000 g of a citrate salt was introduced into the solution to act as a dispersant. The solution was maintained at 50 °C and pH 10 while stirring for 1 h. The specific reaction equation is as follows:

FeCl_2_ + FeCl_3_ + 8NH_4_OH→Fe_3_O_4_ + 8NH_4_Cl + 4H_2_O

After cooling to room temperature, the obtained magnetic nanoparticles were washed alternately with ethanol and water for 3 to 5 cycles. The Fe_3_O_4_ nanoparticles were subsequently placed in a vacuum chamber at 50 °C and dried for 720 min. After drying, the bulk samples were removed and allowed to cool. The samples were then ground into a powder and stored in a hermetically sealed container. As illustrated in Figure 1, Fe_3_O_4_ magnetic nanofluids were prepared using a two-step method, with sodium citrate added as a dispersant to enhance stability. Sodium citrate and Fe_3_O_4_ were mixed and added to pure water to prepare nanofluids at concentrations of 0.025 wt%, 0.050 wt%, 0.075 wt%, 0.100 wt%, 0.125 wt%, and 0.150 wt%. The mixture was subjected to ultrasonic oscillation, with samples collected every 5 min to allow the foam generated by the ultrasonic treatment to settle. After a total ultrasonic oscillation of 40 min, a stable black nanofluid was obtained.

### 2.2. Characterization

Table 1 shows the information of the main instrument manufacturers.

**Figure 2:**
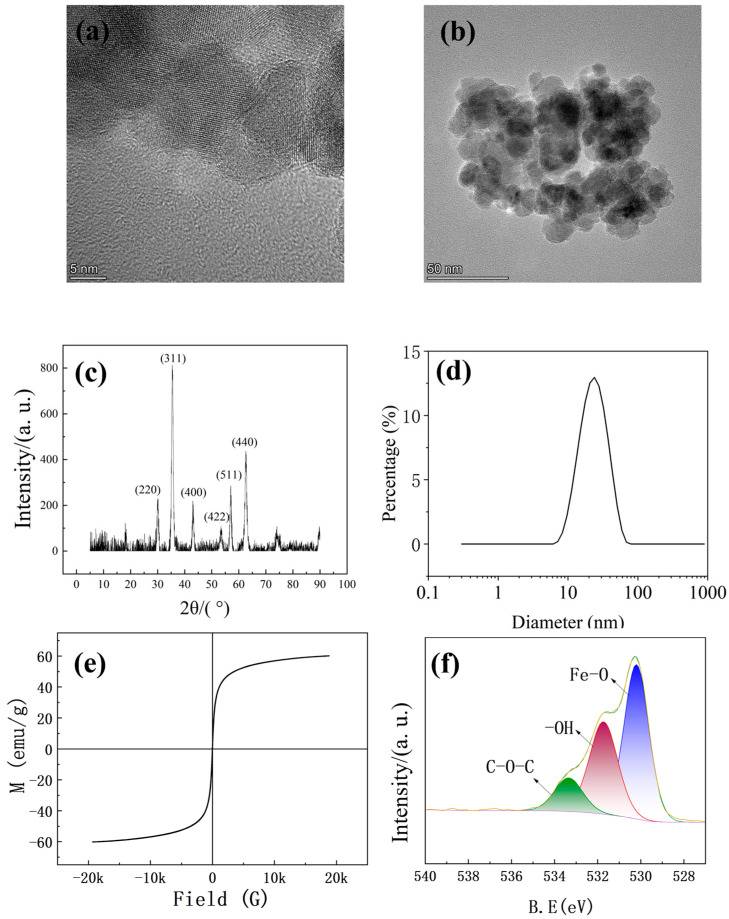
Fe_3_O_4_ material characterization: (**a**) High-magnification Fe_3_O_4_ electron microscope image; (**b**) low-magnification Fe_3_O_4_ electron microscope image; (**c**) XRD diffraction pattern; (**d**) DLS diagram; (**e**) hysteresis loop; (**f**) XPS diagram.

Figure 2 presents the characterization of the prepared Fe_3_O_4_ composite material, with the morphology analyzed using high-resolution transmission electron microscopy (TEM) in Figure 2a,b. The low-magnification electron microscope image, with a scale bar of 5 nm, reveals the aggregation of quasi-spherical nanoparticles composed of irregular subunits ranging from 5 to 15 nm, demonstrating a controlled particle size distribution. Figure 2c shows the X-ray diffraction (XRD) pattern of the sample. The average crystalline grain size d can be calculated using the Scherrer equation, as Equation (1) shows:

(1) $$d=\frac{0.9\lambda }{\beta \cos\theta },$$

where d is the average grain size of the crystals in nanometers; λ is the wavelength of the X-ray, taken as 0.15406 nm, and the full width at half maximum β corresponds to the diffraction angle θ of the Fe_3_O_4_ nanoparticles (311) crystal plane, where the diffraction angle 2θ is 35.45° and the full width at half maximum β is 0.6886°. The average particle size of the Fe_3_O_4_ nanoparticles is approximately 21.25 nm.

As illustrated in Figure 2d, the dynamic light scattering (DLS) measurement shows that the mean particle size of the sample is 21.23 nm.

Figure 2e shows the hysteresis loop. The results indicate that the saturation magnetization of Fe_3_O_4_ is 60.19 emu/g, suggesting that Fe_3_O_4_ exhibits superparamagnetism, influenced by disordered spins and the size of the nanoparticles. In this study, sodium citrate was employed to coat the surface of Fe_3_O_4_ nanoparticles, which suppressed oxidation and preserved bulk-like spin alignment, thereby yielding magnetite with a high saturation magnetization. Figure 2f presents XPS data regarding the surface chemistry and elemental make-up of the sample, confirming Fe_3_O_4_ as the dominant phase, as evidenced by the features in the O1s and C1s regions.

### 2.3. Experimental Setup

**Figure 3:**
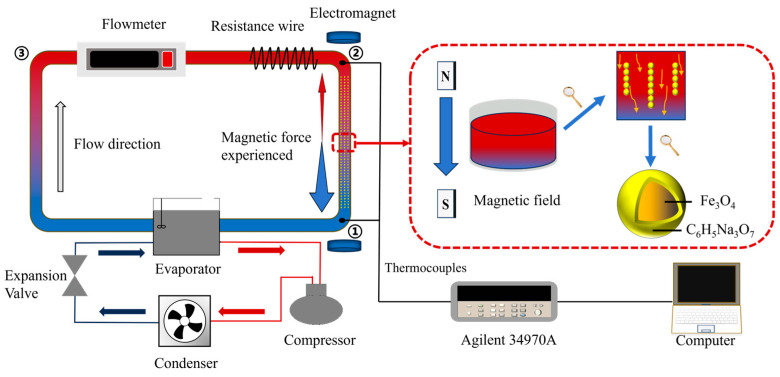
Experimental device.

The experimental setup consists of three sections: the testing section, the cooling section, and the data acquisition section. The experimental setup is shown in Figure 3. The experimental testing section consists of an aluminum tube connected end-to-end. This tube has an outer diameter of 8 mm and an inner diameter of 6 mm, thereby forming a closed pipeline. The length of the pipeline in the magnetic field region is fixed at 10 cm, while its total length can be modified by substituting different aluminum tubes. The closed pipeline is divided into four sections: the heating section, the velocity measurement section, the magnetic field section, and the cooling section. In the heating section, the tube is uniformly wrapped with resistance wire, enabling adjustable heating power. When current is applied, the temperature of the resistance wire rises, resulting in convection heat transfer with the flowing Fe_3_O_4_ magnetic nanofluid inside the tube. The heating power is regulated by adjusting the power supply to meet the experimental requirements. In the heated section, the length is 15 mm. The external heating surface area of the tube is 0.000377 m^2^, and the internal heat transfer area is 0.000282 m^2^. The velocity measurement section employs a KEYENCE flowmeter to measure the instantaneous volume flow velocity of the magnetic nanofluid.

The magnetic field section generates a horizontally uniform magnetic field aligned with the axial direction of the pipeline using an electromagnet. A Gaussmeter is used to quantify the magnetic field, which is then tuned from 0 to 150 mT by adjusting the power supply to meet experimental needs. The cooling section uses a constant temperature water bath to provide continuously circulating cooling water at 2 °C, with an adjustable flow velocity to regulate temperature in the cooling section and ensure it meets experimental demands. Additionally, the exposed sections of the experimental testing component are wrapped with fiberglass to minimize heat loss. The data acquisition section includes thermocouples, the KEYENCE flowmeter, an Agilent 34970A, and a computer, which are used to measure nanofluid temperatures at position ① before heating and at both ends of the uniform magnetic field region at positions ② and ③.

### 2.4. Data Processing and Uncertainty Analysis

Under a temperature gradient, the Fe_3_O_4_-based magnetic nanofluid displays spatially varying magnetization as a result of the thermomagnetic effect. This nonuniform magnetization generates a gradient magnetic force in the gradient field, thereby driving the spontaneous flow of the fluid. The difference in magnetization intensity of the magnetic nanofluid under the influence of the external magnetic field produces a net magnetic driving force $F_{R}$ that drives the fluid from point ② to point ①. The magnetization of the magnetic nanofluid is related to its volume concentration φ, magnetic field strength H, and fluid temperature T. Theoretically, a higher magnetization intensity of the magnetic particles correlates with a greater magnetic force experienced by them.

Magnetization intensity M is expressed as in Equation (2) [22]:

(2) $$M=\frac{\varphi M_{s,p}H}{\left(H_{T}+H\right)}-K(T-T_{0}),$$

where $M_{s,p}$ is the saturation magnetization of the magnetic nanoparticles, which is 60.19 emu/g in the experiment; φ is the volumetric concentration of the magnetic nanofluid; $H_{T}$ is the magnetic field strength when the fluid magnetization intensity equals $M_{S/2}$, approximately 600 mT; H is the magnitude of the externally applied uniform magnetic field; K is the thermomagnetic coefficient; $T_{0}$ is the reference temperature, set here to room temperature at 298 K; T is the temperature of the magnetic nanofluid.

The value of K in the experiment is calculated by Equation (3):

(3) K=0.0028M,

Considering the influence of the temperature variation $\left(T-T_{0}\right)$ on magnetization intensity M and the thermomagnetic coefficient K, magnetization intensity M is expressed as Equation (4) shows:

(4) $$M=\frac{\varphi M_{s,p}H}{\left[1+0.0028\left(T-T_{0}\right)\right]\left(H_{T}+H\right)},$$

The volumetric concentration is the percentage of magnetic particles occupying the volume of the solution, as Equation (5) shows:

(5) $$\varphi =\frac{V_{p}}{V}.$$

Accordingly, the mass concentration is the percentage of magnetic particles occupying the mass of the solution, as Equation (6) shows:

(6) $$\varphi_{m}=\frac{\varphi \rho_{p}}{\rho_{H_{2}O}},$$

where $\rho_{p}$ is the density of the magnetic nanoparticles. $\rho_{H_{2}O}$ is the density of water.

The difference in magnetization ΔM between the hot and cold ends refers to the difference in the magnetization of the magnetic nanofluid at points ① and ② in the loop shown in Figure 2. This difference in magnetization is the driving force for the self-circulation of magnetic nanofluid. Theoretically, the larger the difference in magnetization, the greater the gradient of magnetization in the magnetic field section. As a result, the magnetic driving force $F_{R}$ acting on the fluid increases, leading to a higher flow velocity. The difference in magnetization ΔM is expressed as Equation (7).

(7) $$\Delta M=M_{c}-M_{h},$$

In the experiment, it is necessary to convert the instantaneous volumetric flow velocity measured by the flowmeter into the instantaneous flow velocity. The instantaneous flow velocity v of the nanofluid in the loop is expressed as Equation (8).

(8) $$v=\frac{4Q}{\pi d^{2}},$$

where Q is the instantaneous volumetric flow velocity; d is the inner diameter of the circular pipe.

The heat transfer power between the magnetic nanofluid and the heating section of the pipeline is expressed as Equation (9).

(9) $$P_{l}=P-P_{a},$$

where P is the total heat dissipation power of the heating section (W), P = 9.0 W; $P_{a}$ is the heat dissipation power through the air at the heating section (W); $P_{a}$ is expressed as Equation (10).

(10) $$P_{a}=K_{a}(T_{2}-T_{0}),$$

where $T_{2}$ is the temperature at the heating section when the system temperature is stable with the pipeline filled with magnetic nanofluid (°C); $T_{0}$ is the ambient temperature, where $T_{0}$ = 29.413, °C); $K_{a}$ is the heat transfer coefficient between the outer surface of the pipeline and the air (W/K), expressed as Equation (11).

(11) $$K_{a}=\frac{P}{T_{2,H_{2}O}-T_{0}},$$

where $T_{2,H_{2}O}$ is the temperature at the heating section when the system temperature is stable with the pipeline filled with water at 58 °C.

By substituting Equations (10) and (11) into Equation (9), we can obtain the calculation formula for the heat conduction power of the magnetic nanofluid and the heating section of the pipeline $P_{l}$, expressed as Equation (12)

(12) $$P_{l}=P-\frac{P(T_{2}-T_{0})}{T_{2,H_{2}O}-T_{0}}.$$

(13) $$K_{l}=\frac{P(T_{2,H_{2}O}-T_{0})-P(T_{2}-T_{0})}{(T_{2,H_{2}O}-T_{0})(T_{2}-T_{3})A}.$$

The Reynolds number is calculated by Equation (14).

(14) Re=ρvLμ.

where ρ is the density of the magnetic nanofluid; because its mass concentration is very low, it is approximated as that of water, 1000 kg/m^3^. v is the flow velocity of the magnetic nanofluid. L is the characteristic length of the conduit, which is 0.006 m in this experiment. μ is the dynamic viscosity of the magnetic nanofluid, measured under various conditions using a Brookfield DV3T rotational viscometer.

The Nusselt number is calculated by Equation (15).

(15) $$Nu=\frac{hL}{k_{nf}}.$$

where h is the convective heat transfer coefficient; $k_{nf}$ is the thermal conductivity of the magnetic nanofluid, approximated as that of water.

The thermal conductivity of the water-based Fe_3_O_4_ magnetic nanofluid $k_{nf}$ is calculated by Equation (16) [36].

(16) $$k_{nf}=k_{bf}{\left(1+10.5\varphi \right)}^{0.1051}.$$

where $k_{bf}$ is the thermal conductivity of the base fluid.

Uncertainty in the experimental data may arise from measurement errors of quantities such as temperature and flow velocity. The volume of the nanofluid is measured using a graduated cylinder with an accuracy of 1 mL. The accuracy of the thermocouples is 0.1 °C. The accuracy of the power supply is 0.01 W. The flow meter has an accuracy of 0.001 mL/s. The ruler used to measure the inner diameter of the circular pipe has an accuracy of 0.1 mm.

The error propagation formulas for the volume concentration, viscosity, instantaneous flow velocity, magnetization intensity, and heat transfer coefficient of the nanofluid are presented as follows:

(17) $$\frac{\delta \varphi }{\varphi }=\sqrt{{\left(\frac{\delta V}{V}\right)}^{2}}$$

(18) $$\frac{\delta v}{v}=\sqrt{{\left(\frac{\delta Q}{Q}\right)}^{2}+{\left(\frac{\delta d}{d}\right)}^{2}}$$

(19) $$\frac{\delta \Delta M}{\Delta M}=\frac{\delta M}{M}=\sqrt{{\left(\frac{\delta H}{H}\right)}^{2}+{\left(\frac{\delta T}{T}\right)}^{2}+{\left(\frac{\delta \varphi }{\varphi }\right)}^{2}}$$

(20) $$\frac{\delta K_{l}}{K_{l}}=\sqrt{{\left(\frac{\delta P}{P}\right)}^{2}+{\left(\frac{\delta T}{T}\right)}^{2}}$$

Based on the error propagation formulas, the relative errors are as follows: the volume concentration is 10%, the magnetization intensity is approximately 10%, the instantaneous flow velocity is 3.91%, and the heat transfer coefficient is 10%.

## 3. Results and Discussion

### 3.1. Influence of Pipeline Length

**Figure 4:**
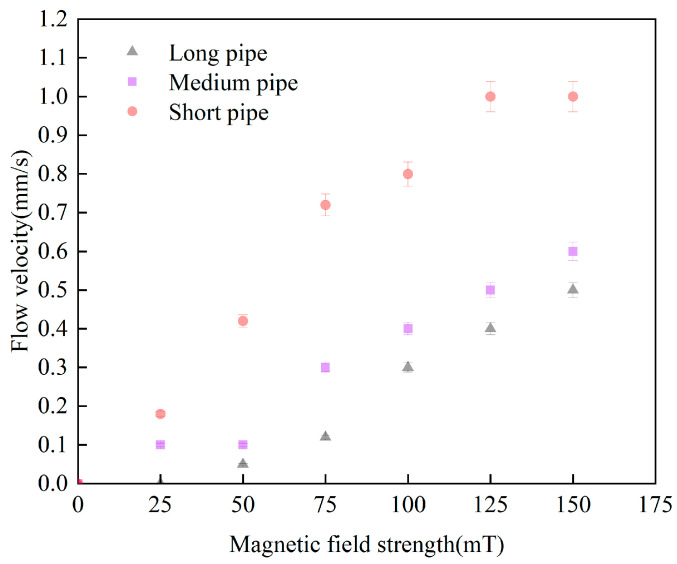
The influence of pipeline length on flow velocity.

This experiment investigated the effect of pipeline length on the flow velocity of magnetic nanofluid at different magnetic field strengths, as shown in Figure 4. The total lengths of the short, medium, and long pipes were 60 cm, 80 cm, and 100 cm, respectively. The flow velocity of fluids with different concentrations decreases as the pipeline length increases. At an applied magnetic field strength of 75 mT, the flow velocity in the long pipe was 0.12 mm/s, compared to 0.30 mm/s in the medium pipe and 0.72 mm/s in the short pipe, indicating reductions of 60% and 83%, respectively. This reduction is attributed to the increased pipeline length, which significantly raises frictional resistance. According to the Hagen–Poiseuille law, flow resistance is proportional to pipeline length, while the magnetic driving force generated by the thermomagnetic effect only acts on the fixed segment of the pipeline with the magnetic field applied, remaining relatively constant regardless of the total pipeline length. Therefore, increasing the pipeline length sharply increases frictional resistance relative to the magnetic driving force. Additionally, for the same temperature difference at the hot and cold ends, a longer total pipeline length results in a lower average temperature of the unmagnetized pipe segment. The decrease in temperature significantly increases fluid viscosity, further raising overall flow resistance. Overall, increasing the pipeline length raises both frictional resistance and overall viscosity, ultimately leading to a substantial decrease in flow velocity. Consequently, a pipeline length of 60 cm was chosen for subsequent experimental studies.

### 3.2. Thermomagnetic Convection Characteristics

**Figure 5:**
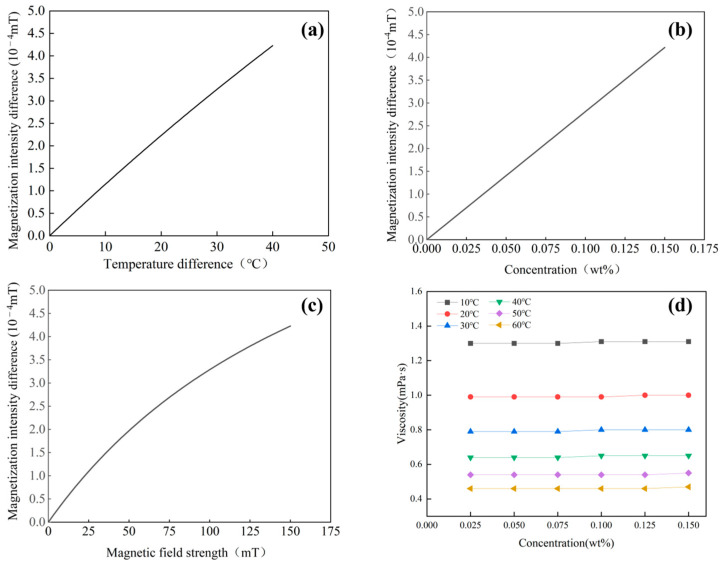
(**a**) The influence of temperature on magnetization intensity difference; (**b**) the influence of concentration on magnetization intensity difference; (**c**) the influence of magnetic field strength on magnetization intensity difference; (**d**) the influence of temperature and concentration on viscosity.

Building on theoretical analysis and experimental results, we characterized how temperature difference, particle concentration, and magnetic field strength influence both the magnetization difference and the viscosity of the magnetic nanofluid, as depicted in Figure 5. Figure 5a illustrates the effect of the temperature difference between the hot and cold ends on the magnetization intensity difference at a magnetic field strength of 150 mT. The figure shows that, at a constant magnetic field strength, the magnetization intensity difference in the magnetic nanofluid exhibits a significant positive correlation with the temperature difference. As the temperature difference increases, the magnetization intensity difference demonstrates a clear monotonous increase, rising from 0 to 4.0 × 10^−4^ mT. Figure 5b,c depict how concentration modulates the magnetization difference magnitude of the magnetic nanofluid under a 40 °C temperature gradient and a 150 mT magnetic field, and, conversely, how alterations in magnetic field strength influence these properties when the temperature gradient remains at 40 °C and the particle concentration is fixed at 0.150 wt%. The results indicate that, under a constant temperature difference, the magnetization intensity difference in the magnetic nanofluid at the cold and hot ends exhibits a significant positive correlation with both volume concentration and magnetic field strength. As volume concentration and magnetic field strength increase, the magnetization intensity difference demonstrates a clear monotonous increase, rising from 0 to 4.0 × 10^−4^ mT.

Figure 5d presents the effect of temperature and concentration on the viscosity of the magnetic nanofluid. The results indicate a significant negative correlation between temperature and viscosity, with viscosity markedly decreasing as temperature rises. This occurs because, within the allowable temperature range of the experiment, an increase in temperature enhances particle motion, increases intermolecular distance, and weakens intermolecular attraction, thereby reducing internal friction and subsequently lowering both viscosity and sedimentation rate of the magnetic nanofluid. In comparison with temperature, changes in volume fraction and magnetic field strength have only negligible effects on viscosity. This is primarily attributed to the low volume concentration of the magnetic nanofluid used in the experiment, where the directional alignment of particles and the increased rotational resistance due to the magnetic field have relatively minor effects on the overall flow resistance of the fluid.

**Figure 6:**
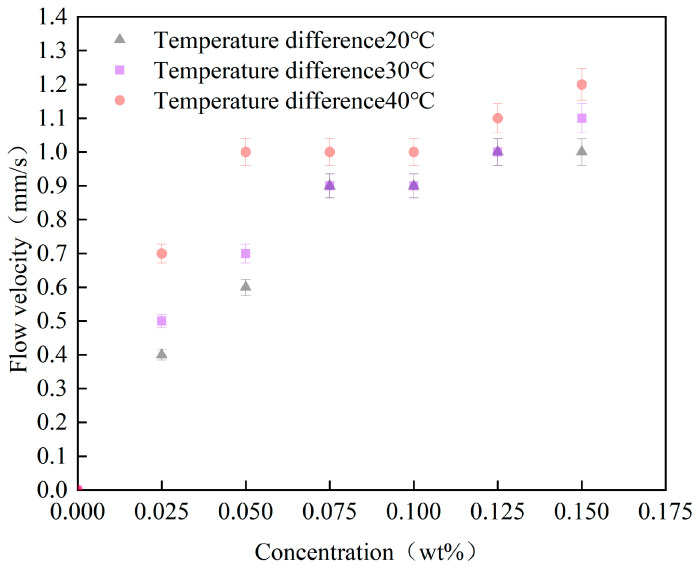
The influence of the temperature difference between the cold and hot ends on flow velocity.

The magnetic field strength was set to 150 mT by adjusting the power of the electromagnet, while the heating power of the electric heating wire was adjusted to maintain both the pipeline temperature and the temperature difference between the hot and cold ends at fixed values. Stable operating flow velocities were then measured. This experiment examined the effects of varying concentrations of Fe_3_O_4_ magnetic nanofluid on flow velocity under different temperature differences between the hot and cold ends, with results presented in Figure 6. As the temperature difference between the two ends of the magnetic nanofluid increases, the flow velocity of fluids with varying concentrations also exhibits an increase. At concentrations of 0.025 wt% and 0.050 wt%, the flow velocity of the magnetic nanofluid at a temperature difference of 40 °C increased by 75% and 40%, respectively, compared to the flow velocity observed at a temperature difference of 20 °C. When the concentration of the magnetic nanofluid exceeds 0.075 wt%, the flow velocity at a temperature difference of 40 °C remains higher than that at 20 °C; however, the increase is diminished, averaging 11%. This occurs because, under constant external magnetic field conditions, the magnetization intensity of the magnetic nanofluid exhibits a negative correlation with temperature. An increase in temperature at the heated end results in a decrease in the magnetization value of the magnetic nanofluid in that region, thereby increasing the magnetic field intensity difference between the hot and cold ends and enhancing the driving force generated by the thermomagnetic effect. Additionally, temperature is the primary factor affecting viscosity. As illustrated in Figure 5d, as temperature increases, the viscosity of the magnetic nanofluid decreases significantly, resulting in reduced flow resistance.

The experiments consolidated all measured data and yielded an empirical correlation Equation (21) for the influence of concentration, temperature difference, and magnetic field strength on the flow velocity of the magnetic nanofluid ν, as follows:

(21) $$\nu =0.594\varphi^{0.02}{\left(\Delta T\right)}^{0.211}H^{0.874}.$$

**Figure 7:**
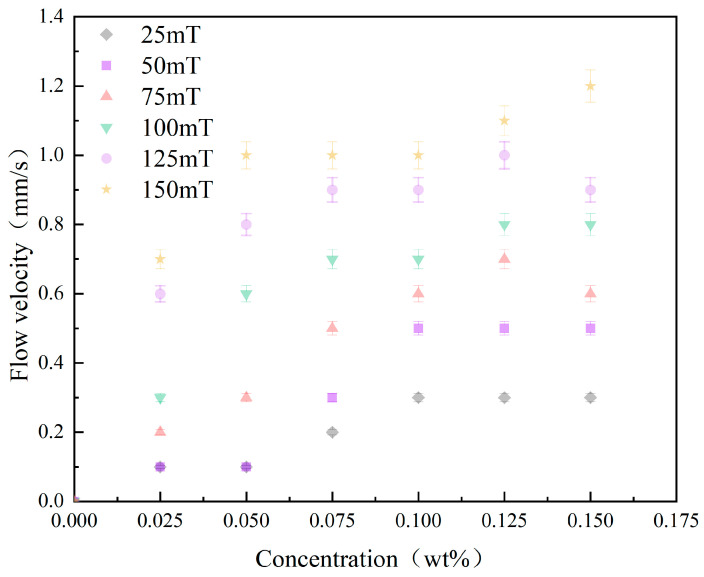
The influence of concentration and magnetic field strength on flow velocity.

**Figure 8:**
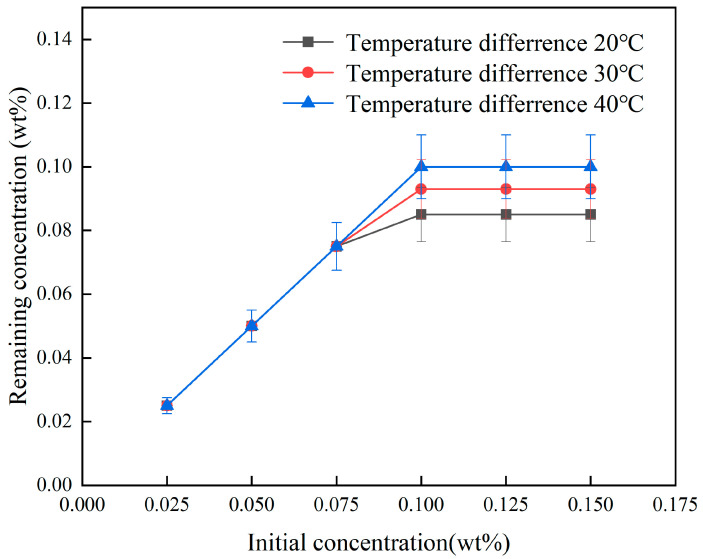
The loss of ferrofluid concentration under a magnetic field strength of 150 mT.

The heating power of the electric heating wire was adjusted to achieve a temperature difference of 40 °C in the pipeline using magnetic nanofluids with varying concentrations. Meanwhile, the power of the electromagnet was modified to measure stable flow velocity under different magnetic field strengths, thereby investigating the effects of fluid concentration and magnetic field strength on the thermomagnetic effect, with results presented in Figure 7. Figure 7 illustrates that the flow velocity of the fluid in the pipe increases with the concentration of magnetic nanoparticles. At a magnetic field strength of 150 mT, the flow velocities of the nanofluids with concentrations of 0.075 wt% and 0.150 wt% were 1.0 mm/s and 1.2 mm/s, respectively, indicating increases of 43% and 71% compared to the baseline flow velocity of 0.7 mm/s for the concentration of 0.025 wt%. A similar significant increasing trend was observed at other magnetic field strengths as well. Increasing the concentration enhances both the saturation magnetization and overall magnetization of the magnetic nanofluid, thereby strengthening the thermomagnetic driving force; the enhancement of the thermomagnetic effect by concentration is comparable to that caused by the temperature difference between the hot and cold ends. However, Figure 7 also indicates that when the concentration of the magnetic nanofluid exceeds 0.075 wt%, the rate of flow increase diminishes. As shown in Figure 8, this phenomenon is attributed to strong magnetic field interactions among the suspended magnetic nanoparticles in the magnetic nanofluid under high magnetic field strength conditions, where the attraction between particles exceeds the dispersive force, causing particles to aggregate and sediment. In a continuous flow system, particle magnetization is the most critical factor affecting separation efficiency [37]. Even under the same magnetic field conditions, particles with lower magnetization are difficult to effectively capture. Rising temperature reduces the magnetization of Fe_3_O_4_ particles, thereby enhancing the stability of the ferrofluid and decreasing the number of particles that undergo aggregation.

Figure 7 illustrates that as the volume concentration of the magnetic nanofluid increases, the flow velocity under different magnetic field strengths also increases. At magnetic field strengths of 75 mT and 150 mT, the flow velocity of the nanofluid with a concentration of 0.100 wt% was 0.6 mm/s and 1.0 mm/s, respectively, indicating increases of 100% and 233% compared to the baseline flow velocity of 0.3 mm/s at 25 mT. At magnetic field strengths of 75 mT and 150 mT, the flow velocity of the nanofluid with a concentration of 0.150 wt% was 0.6 mm/s and 1.2 mm/s, respectively, indicating increases of 100% and 300% compared to the baseline flow velocity of 0.3 mm/s at 25 mT, which is a greater increase than that observed at a concentration of 0.100 wt%. A similar significant increasing trend was observed under other temperature difference conditions as well. This phenomenon is fundamentally due to the superparamagnetism of Fe_3_O_4_ nanoparticles, where an increase in the external magnetic field significantly enhances the magnetization intensity of the magnetic nanofluid, increasing both the magnetization intensity difference and the magnetization gradient, which directly boosts the thermomagnetic driving force. Additionally, an increase in the external magnetic field elevates the thermomagnetic effect, further enhancing the magnetization intensity difference between the high-temperature and low-temperature regions. Conversely, within the concentration range of the Fe_3_O_4_ magnetic nanofluid investigated in this study, changes in magnetic field strength have a minimal effect on viscosity.

**Figure 9:**
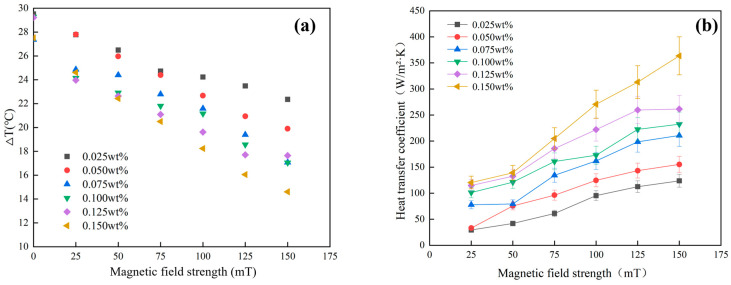
(**a**) The influence of concentration and magnetic field strength on the temperature difference between the hot and cold ends; (**b**) the influence of concentration and magnetic field strength on the heat transfer coefficient.

The electric heating wire’s power was set to 9 W (23,872 W/m^2^), and changes were made to the concentration of the magnetic nanofluid and the magnetic field strength to measure temperatures at points ①, ②, and ③. Subsequently, heat dissipation and the heat transfer coefficient between the magnetic nanofluid and the inner wall of the pipeline were calculated. As illustrated in Figure 9a, as the concentration and magnetic field strength of the magnetic nanofluid increase, the temperature difference between the hot and cold ends gradually decreases. This is attributed to the fact that, as the concentration and magnetic field strength of the magnetic nanofluid increase, the flow velocity of the magnetic nanofluid in the pipeline accelerates, transporting more heat away from the heated area, thereby lowering the temperature at point ②. The shortened flow time of the magnetic nanofluid before entering the water bath results in decreased heat dissipation to the air, leading to a slight increase in temperature at point ③. This ultimately causes a reduction in the temperature difference between the hot and cold ends, while further increases in magnetic field strength and concentration slow down any enhancement in flow velocity.

As indicated in Figure 9b, as the magnetic field strength increases, the heat dissipation between the inner wall of the pipeline and the magnetic nanofluid of varying concentrations also increases. At a constant magnetic field strength, higher concentrations of the magnetic nanofluid lead to increased heat dissipation and the heat transfer coefficient. Additionally, Figure 9b shows that higher concentrations correspond to a larger increase in the heat transfer coefficient as the magnetic field strength rises. The increase in the heat transfer coefficient at a magnetic field strength of 150 mT, compared to that at 25 mT, varies by magnetic nanofluid concentration as follows: 94 W/m^2^·K, 122 W/m^2^·K, 133 W/m^2^·K, 131 W/m^2^·K, 147 W/m^2^·K, and 243 W/m^2^·K.

**Figure 10:**
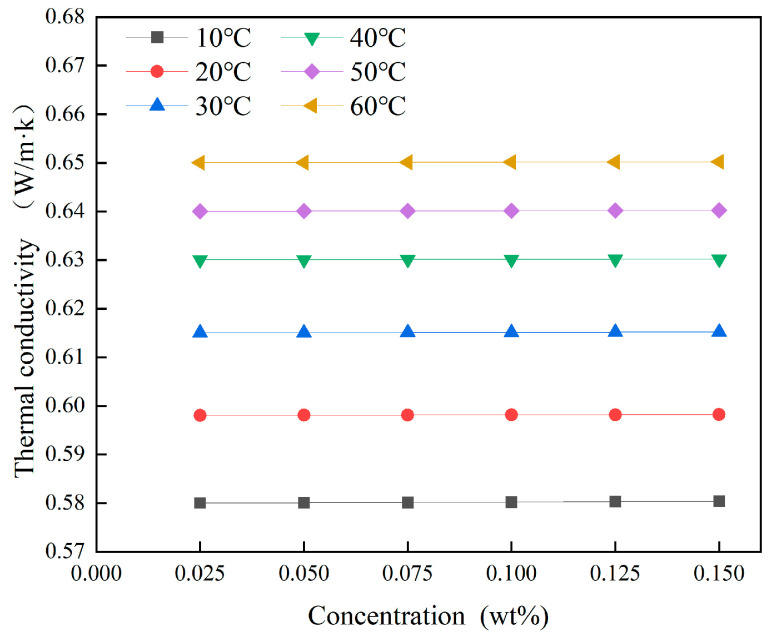
Influence of temperature and concentration on the thermal conductivity of magnetic nanofluids.

Using Equation (16), the thermal conductivities of Fe_3_O_4_ magnetic nanofluid at different concentrations were calculated across a range of temperatures. The results, shown in Figure 10, indicate that temperature plays the dominant role in determining the thermal conductivity of the nanofluids in this study.

**Figure 11:**
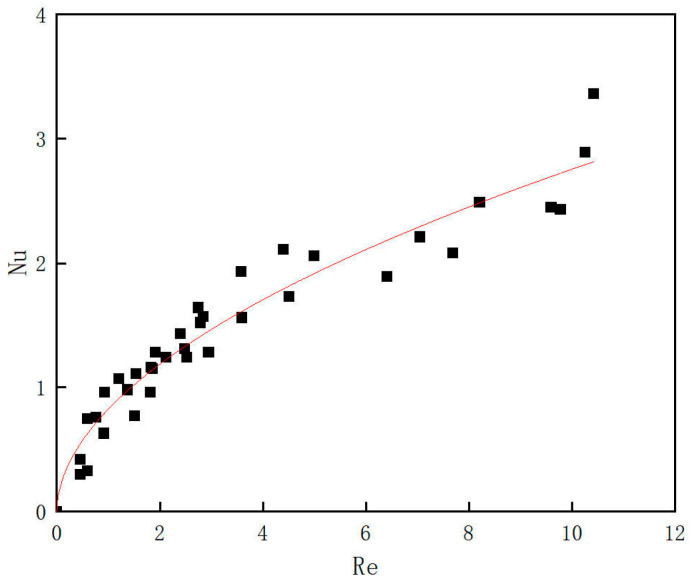
The relationship between the Nusselt number and the Reynolds number.

Figure 11 shows the relationship between the Reynolds number and the Nusselt number; the fitted curve is expressed as Equation (22):

(22) Nu=0.825 Re0.524.

From Figure 11, the convective heat transfer coefficient between the pipe inner wall and the magnetic nanofluid increases with the Reynolds number. At the minimum Reynolds number of 0.46, the Nusselt number is 0.30; the flow is a strongly viscosity-dominated creeping flow, and heat transfer is almost entirely governed by conduction. At the maximum Reynolds number of 10.42, the Nusselt number is 3.36, which approaches the theoretical constant of 3.66 for fully developed laminar flow with a constant wall temperature boundary condition. In this regime, the flow is viscosity-dominated laminar; the overall velocity field is fully developed, while the thermal field is close to, but does not fully reach, the classical condition.

### 3.3. Comparison with Other Research

The magnetic nanofluid removes heat from the heating section via flow. A higher flow velocity leads to a larger heat transfer coefficient between the magnetic nanofluid and the tube wall, resulting in a more pronounced cooling effect. Table 2 presents the maximum flow velocity of different magnetic nanofluid circulation devices under their respective experimental conditions. The experimental results indicate that although the flow velocity achieved in this study is relatively low, this work realizes the self-circulation of magnetic nanofluid at a low concentration by employing a uniform magnetic field with low magnetic induction intensity and using magnetite as the magnetic nanofluid, which features lower application cost and higher Curie temperature. Compared with other studies, this work employs a more cost-effective magnetite nanofluid and uses a uniform magnetic field. Relative to Lian’s experimental results [22], our experiments achieve a higher flow velocity of 1.2 mm/s under a lower magnetic field strength. Compared with Yuhiro’s experimental results [30], the magnetic field strength used here is similar, but the flow velocity is lower than Yuhiro’s 1.79 mm/s. Relative to Fumoto’s simulation results [20], our flow velocity is lower than 10 mm/s, whereas the magnetic field strength employed is smaller. Compared with Dalvi’s simulation results [25], the flow velocity is lower than 17 mm/s, but again, the magnetic field strength applied here is smaller. The cooling purpose is successfully achieved, and a scheme for magnetic nanofluid self-circulation cooling under a uniform magnetic field is explored, thereby providing an important reference for the design optimization of novel liquid cooling systems.

### 3.4. Comparison of Influencing Factors

This study employs an L9(3^4^) orthogonal array to investigate the effects of four factors—pipeline length, magnetic nanofluid concentration, temperature difference between the hot and cold ends, and magnetic field strength—on thermomagnetic driving flow velocity. Each factor is assigned three levels, and the contribution weights of each factor to the flow velocity are quantified using range analysis to elucidate the primary control mechanisms under the coupling of multiple parameters. Table 3 details the orthogonal experiment, which comprises a total of nine test conditions: pipeline lengths of 60 cm, 80 cm, and 100 cm; magnetic nanofluid concentrations of 0.025 wt%, 0.050 wt%, and 0.075 wt%; temperature differences between the hot and cold ends of 20 °C, 30 °C, and 40 °C; and magnetic field strengths of 50 mT, 100 mT, and 150 mT. The significance of each factor is assessed through range R calculations, where a larger range value indicates a greater influence of that factor on the flow velocity; the range is calculated using Equation (23).

(23) $$R=Max\left(\bar{K_{i}}\right)-Min\left(\bar{K_{i}}\right).$$

In the formula, $\bar{K_{i}}$ represents the average flow velocity at the same level of each factor, that is, the mean value.

**Figure 12:**
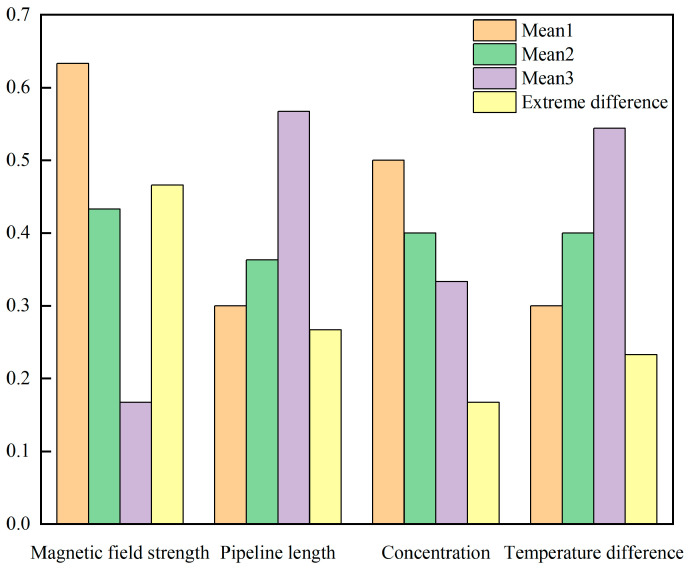
Results of orthogonal experiment.

Figure 12 displays the results of the orthogonal experiments examining the influence of four factors on the flow velocity enhancement of Fe_3_O_4_ magnetic nanofluid. The calculated extreme difference values for pipeline length, concentration, temperature difference between the hot and cold ends, and magnetic field strength are 0.267, 0.167, 0.233, and 0.466, respectively. Magnetic field strength is the primary factor that directly influences the magnetization of the magnetic nanofluid. Increasing the magnetic field strength significantly enhances magnetization, thereby boosting the driving force F_R_ generated by the thermomagnetic effect. Within the scope of this study, the influence of magnetic field strength on the viscosity of Fe_3_O_4_ nanofluid is relatively minor, rendering the effects of changes in magnetic field strength on flow resistance negligible. Magnetic field strength is approximately linearly positively correlated with magnetization intensity, which directly determines the magnitude of the magnetic driving force and consequently leads to a significant enhancement in flow velocity. Pipeline length is a secondary factor influencing flow velocity. An increase in pipeline length results in significantly higher flow resistance, while the magnetic driving force acts only on the fixed segment of the pipeline that is within the magnetic field. According to the Hagen–Poiseuille law, resistance loss is proportional to pipe length. Furthermore, the average temperature in the non-magnetic field segments is closer to room temperature and lower than that in the magnetic field segments, which results in increased viscosity and consequently leads to a significant increase in resistance and a considerable decrease in flow velocity. The temperature difference between the hot and cold ends influences the flow velocity by enhancing the magnetic driving force and reducing viscosity. However, because the temperature differences involved in the experiments are far from the Curie temperature of Fe_3_O_4_, the sensitivity of magnetization to temperature changes is low, resulting in limited enhancement of the driving force. Increasing the concentration of the magnetic nanofluid enhances the number of Fe_3_O_4_ particles within the fluid, thereby improving overall magnetization. Similar to magnetic field strength, magnetic nanofluid concentration also directly affects its magnetization. Although increasing the concentration of the magnetic nanofluid enhances its magnetization, at higher magnetic field strengths, highly concentrated magnetic nanofluids are prone to sedimentation, diminishing their effect. The research results indicate that magnetic field strength is the dominant factor due to its direct regulation of the magnetic driving force, while pipeline length is secondary, as it amplifies resistance. The temperature difference between the hot and cold ends has a weaker influence because the Curie temperature is much higher than the experimental range, and the influence of concentration is minimized due to sedimentation issues.

## 4. Conclusions

In this study, sodium citrate was employed as a surfactant to disperse Fe_3_O_4_ magnetic nanoparticles. Fe_3_O_4_ magnetic nanofluid exhibits superparamagnetism. The effects of magnetic field strength, magnetic nanofluid concentration, temperature difference between the hot and cold ends, and pipeline length on thermomagnetic effect and heat transfer characteristics. The following key conclusions were drawn:

(1) It has been established that as magnetic field strength and magnetic nanofluid concentration increase, the magnetization difference in the magnetic nanofluid increases, while viscosity remains largely unaffected by magnetic field strength. As the temperature difference between the hot and cold ends increases, the magnetization difference in the magnetic nanofluid decreases, along with the viscosity of the magnetic nanofluid.
(2) Under specific conditions, Fe_3_O_4_ magnetic nanofluid can achieve self-circulation within a closed pipeline. The maximum stable circulation parameters identified in the experiment include a magnetic field strength of 150 mT, a magnetic nanofluid concentration of 0.150 wt%, and a temperature difference of 40 °C in a short pipe, yielding a maximum circulation flow velocity of 1.2 mm/s.
(3) The circulation flow velocity increases with magnetic field strength, magnetic nanofluid concentration, and temperature difference between the hot and cold ends in the magnetic field area, while it decreases with increasing pipeline length. Furthermore, as fluid flow velocity increases, the heat transfer coefficient between the inner wall of the pipe and the fluid also increases, enhancing the heat dissipation performance of the cooling device in the heating section.
(4) The orthogonal experiment indicated that the priority of factors on the thermomagnetic effect is ranked as magnetic field strength > pipeline length > temperature difference > magnetic nanofluid concentration.

## Figures and Tables

**Table 1:** Manufacturer’s information.

Instrument	Model	Manufacturer	City	Country
TEM	Tecnai F20	FEI	Hillsboro, Oregon	USA
XRD	SmartLab SE	Rigaku	Takatsuki, Osaka Prefecture	Japan
DLS	Zetasizer Nano ZS90	Malvern	Malvern	UK
XPS	K-Alpha	Thermo Fisher Scientific	Waltham, Massachusetts	USA
Flowmeter	FD-XA1, FD-XS8, FD-XC8M	KEYENCE	Tokyo	Japan
DAU	34970A	Agilent	Santa Clara, California	USA

**Table 2:** Comparison with other research.

	Magnetic Nanofluid	Magnetic Field Types and Intensity	Temperature Difference or Heat Load	Flow Velocity
Lian [22]	Mn-Zn ferrite particles in kerosene	Non-uniform magnetic field, 500 mT	5000 W/m^2^	0.75 mm/s
This study	0.150 wt%Fe_3_O_4_ in water	Uniform magnetic field, 150 mT	40 °C	1.2 mm/s
Yuhiro [30]	Mn-Zn ferrite particles in kerosene	Non-uniform magnetic field, 136 mT	5000 W/m^2^	1.79 mm/s
Fumoto [20]	Mn-Zn ferrite particles in kerosene	Non-uniform magnetic field, 406 mT	40 °C	10 mm/s
Dalvi [25]	5% Gadolinium in kerosene	Non-uniform magnetic field, 500 mT	40 °C	17 mm/s

**Table 3:** Orthogonal experimental design.

Factors	Pipeline Length	Concentration	Temperature Difference	Magnetic Field Strength
Experiment 1	100 cm	0.075 wt%	40 °C	150 mT
Experiment 2	100 cm	0.050 wt%	30 °C	100 mT
Experiment 3	100 cm	0.025 wt%	20 °C	50 mT
Experiment 4	80 cm	0.075 wt%	30 °C	50 mT
Experiment 5	80 cm	0.050 wt%	20 °C	150 mT
Experiment 6	80 cm	0.025 wt%	40 °C	100 mT
Experiment 7	60 cm	0.075 wt%	20 °C	100 mT
Experiment 8	60 cm	0.050 wt%	40 °C	50 mT
Experiment 9	60 cm	0.025 wt%	30 °C	150 mT

## Data Availability

The original contributions presented in this study are included in the article. Further inquiries can be directed to the corresponding author.
